# Stratified approaches for predicting immunometabolic depression-associated features through biomarker-symptom clustering, network profiling, cutoff definition, and delineation of non-linear trends: A methodological overview

**DOI:** 10.1192/j.eurpsy.2026.10964

**Published:** 2026-07-31

**Authors:** A. Hallab

**Affiliations:** 1 Charité Universitätsmedizin Berlin, Berlin, Germany; 2 Harvard Medical School, Boston, United States; 3 Center for global epilepsy - University of Oxford, Oxford, United Kingdom

## Abstract

**Introduction:**

Depression presents a global public health challenge owing to increased morbidity and mortality rates. Dysregulations in the metabolic and immunological homeostasis are associated with a higher risk of therapy resistance and chronicity.

**Objectives:**

The abstract presents a methodological approach for a better risk stratification, particularly in older multiethnic populations.

**Methods:**

The statistical approach uses data collected in the frame of a single-site study, including community-dwelling middle-aged and older adults from North Texas. All participants with and without current depression were eligible. The Geriatric depression scale (GDS) was dichotomized into four items related to features commonly described in depression.

The associations between variables were assessed using Spearman’s correlation and plotted into a network, and only significant coefficients with |r| at least 0.1 were visualized. The edges were weighted based on the strength of the correlation coefficient. The nodes presented the variables. The distance between nodes was weighted based on the strength of the correlation between the variables. Variables establishing direct edges between the immunometabolic and depression-specific features were further explored in adjusted regression models. Generalized additive models and splines were used. Depression- and age-specific stratifications were performed.

**Results:**

The networks and clusters allowed highlighting significant actors in the immune-metabolic and depression-related features constellation. While linear models explained some associations, clinical cutoffs were practical approaches to overcome the non-linearity of some associations.

**Conclusions:**

Stratified network-based methods provided a deeper understanding of the association between aging, depression, and low-grade inflammation, and symptom clustering outperformed the globalistic approach in understanding the immunometabolic depression.

**Acknowledgment:**

Research reported on this publication was supported by the National Institute on Aging of the National Institutes of Health under Award Numbers R01AG054073, R01AG058533, R01AG070862, P41EB015922, and U19AG078109.

**Disclosure of Interest:**

None Declared

